# Integrating Effects of Human Physiology, Psychology, and Individual Variations on Satiety–An Exploratory Study

**DOI:** 10.3389/fnut.2022.872169

**Published:** 2022-04-27

**Authors:** Dongdong Ni, Heather E. Smyth, Daniel Cozzolino, Michael J. Gidley

**Affiliations:** Centre for Nutrition and Food Sciences, Queensland Alliance for Agriculture and Food Innovation, The University of Queensland, Brisbane, QLD, Australia

**Keywords:** satiety, human physiology, psychology, individual variations, food factors

## Abstract

Satiety can influence food intake, and as a consequence has the potential to affect weight and obesity. Human factors such as physiology and psychology are likely to be important in determining satiety. However, it is not well-understood how these factors (individual variations) alone or combined contribute to satiety feelings. In addition, there have been limited or no attempts to use a holistic approach to evaluate satiety. In this study, three plant-based foods were used as mid-morning snack for 52 participants to evaluate satiety response (during three consecutive days, one-day-one-food type). The foods were served *ad libitum* until participants felt comfortably full prior to satiety monitoring. The study explored diverse human factors (*n* = 30) that might contribute to satiety including those related to oral physiology, metabolic factors, body composition and psychology. It identified important variables for satiety as well as the interactions among them and the influences of age, gender, and low satiety phenotype (consistently lower reported fullness scores) on satiety. Overall, combinations of factors rather than individual ones contributed to self-reported satiety. Food factors (e.g., type, composition) had limited effects, but there were only three types used in the study. The combination of metabolic factors [respiratory quotient, age, and body energy usage type (e.g., carbohydrate or fat)], oral sensitivity & processing, personality traits (agreeableness, conscientiousness, and neuroticism), and eating behavior (e.g., emotional and external eating) were the most important for explaining individual satiety responses. Older participants had significantly higher reported satiety than younger participants, associated with significant differences in oral physiology, increased body fat, and mature psychological characters. Moreover, different satiety phenotypes had significant differences in relationships with body fat, oral physiology, personalities, food neophobia, and eating behaviors. The results of this study indicate that much greater insights into the factors determining satiety responses can be obtained by combining multiple food and human physiological and psychological characteristics. This study used more diverse measures of individual variation than previous studies of satiety and points the way toward a more holistic approach to understanding the (control of) perceptions of fullness at both individual and group levels.

## Introduction

Satiety, defined as the perceived feeling of fullness, is one of the driving forces that controls eating episodes (1–3). Satiety plays a role in modulating the daily meal frequency/size and can be a key factor in controlling food intake. This fact is important in developing strategies to manage nutrition and in particular body condition and weight (1–3).

Many factors have been reported that contribute to explaining and controlling satiety including both external environmental cues (e.g., food cue stimulations, daily diet schedules, and social needs) and inherent human factors (e.g., gender, age, physiology, psychology) (4–11). Currently, human factors such as body composition and metabolic factors are usually considered the most important. For example, fat free mass (FFM) and resting metabolic rate (RMR) have been found to be strongly associated with daily food or energy intake, and hunger (4–6). In addition, both oral sensory and processing are also considered to have effects on both satiety and food intake. It has been reported that longer oral processing decreases food intake which may be associated with greater oral sensory exposure and slower eating rate (12–15). This fact may be related with oral and gastrointestinal sensing, hormonal responses, and the physical structure of the food bolus as reported by several authors (12, 14–17).

It has been reported that not only human physiology but also psychology plays an important role in the control and modulation of appetite (satiety) (7–11). Personality traits have also been included in recent studies relating to food choice and eating behavior (18–21). For example (20) reported that personality traits like higher openness and extraversion (related to greater intellect, curiosity, and social engagement) and conscientiousness (related to discipline) might be positively associated with greater intake of plant foods such as fruits and vegetables. However, the relationships between psychological factors (e.g., cognition and memory) and appetite, as well as the effects of interactions between human physiology and psychology on satiety are not well-known. In addition, recent studies have highlighted the effect of age, gender, and low satiety phenotype on satiety (22–26). The low satiety phenotype (27–29) is defined as individuals who appropriately recognized their appetite sensations but reported low appetite values and low changes in appetite sensation. However, the main drivers (involving human physiology and psychology) contributing to these individual effects (e.g., satiety perception phenotypes) are not well-known. Overall, it is clear that diverse factors should be considered to influence individual perceptions of satiety. Consequently, a comprehensive framework is needed that includes and combines multiple and diverse human factors together with food factors to explain satiety.

This study aimed to explore the variation and identify the contribution of a large number of human variables (*n* = 30) including physiological and psychological factors as well as effects of individual variance such as age, gender, and satiety perception phenotype on satiety using three test foods as mid-morning snacks.

## Materials and Methods

### Overall Study Design

**Figure 1:**
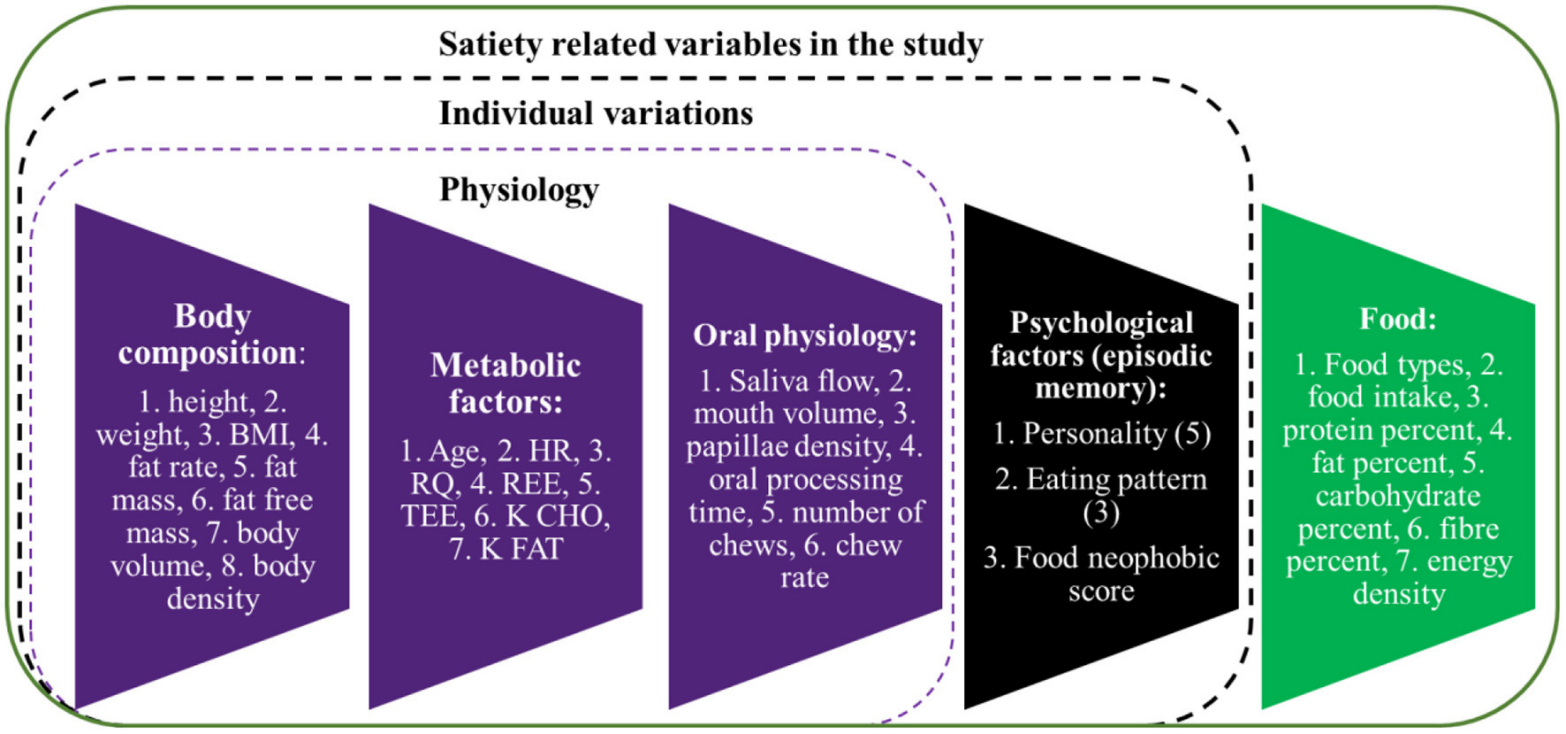
Satiety related variables included in the study. BMI, body mass index; HR, heart rate; RQ, resperatory quotient; REE, resting energy expenditure; TEE, total energy expenditure; K CHO, body energy usage type ratio for carbohydrate; K FAT, body energy usage type ratio for fat.

The study focused on human factors (total 30 variables, Figure 1) including body composition, metabolic, oral physiological, and psychological factors, contributing to satiety. The whole experiment was sub-divided into three parts: (1) satiety sensory experiments, (2) oral physiological and psychological factors measurements, and (3) body composition and metabolic factors measurements.

**Figure 2:**
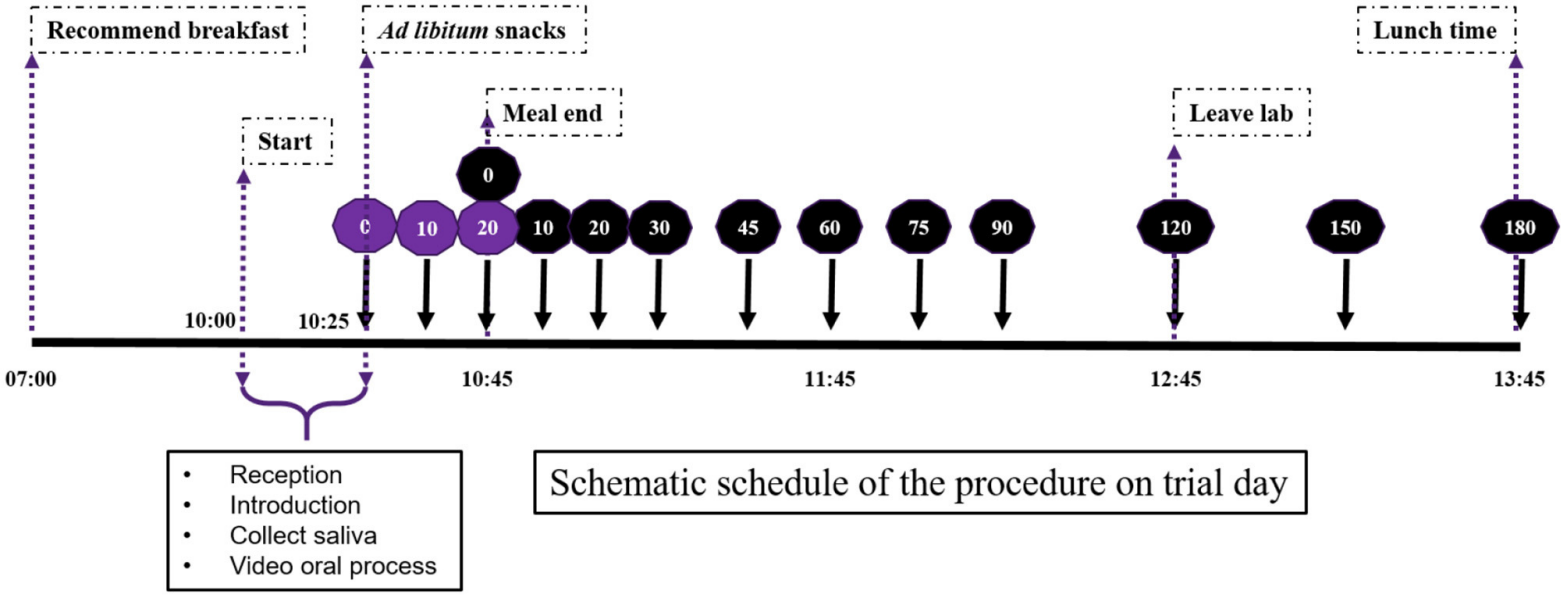
Schematic schedule of the procedure on the satiety sensory trial day. Perceived fullness scores were recorded 0, 10, 20, 30, 45, 60, 75, 90, 120, 150 and 180 mins after food consumption. Purple color coding is during the meal and black is postprandial time points.

Three types of plant-based foods (apple, banana, and avocado) were used as mid-morning snacks for the satiety sensory experiment. This study had a within-subject design. Three foods were served on three different days (1-day-one-food type). In the sensory experiment, snacks were served in *ad libitum* mode for 20 min, and participants were instructed to eat until they felt comfortably full (no food intake quantity was defined). No other food was served to the participants. Therefore, the satiety experiment starting point was the comfortable fullness level, not the traditional same preload food quantity or energy intake. This design was intended to mimic the free-living food intake style of daily life. The timeline (Figure 2) and procedure of the satiety experiment was as described previously (30).

In the oral physiology measurements, participants' unstimulated saliva was collected on three different days as biological replicates for further analysis. The detailed procedure for saliva collection was as described in (31). This research was approved by the Sub-Committee for Human Research Ethics of the University of Queensland Science (approval number: 2019002688).

### Participants

Healthy participants (*n* = 52) were recruited through open advertisement for this study. Detailed participant selection criteria were as follows (1) aged between 18 and 70 years, (2) no oral cavities or dental diseases, (3) no diabetes, (4) not pregnant or lactating, (5) no diagnosed mental diseases like depression, and (6) like eating and no allergy to fruits including apple, banana, and avocado, as described previously (30). All participants attended the satiety sensory experiment and oral physiological and psychological factors measurements. However, due to COVID-19 pandemic restrictions, only 21 participants attended the body composition and metabolic factor measurements. In terms of the groups, individual differences including age, gender, satiety perception phenotype were considered as groupings. The participants aged over 47 were assigned to the old group (16), with age from 18 to 46 being the young group (32). This was a convenient but arbitrary age for the current participant cohort within the range of middle age during which human physiology (e.g., hormones, metabolism) and psychology may change with potential influence on satiety. In gender groups, male vs. female was 21 vs. 31. Individuals were classified as low, intermediate, or high satiety perceiving groups, defined as those individuals that report high [1,500–2,500 total area under satiety curve (AUC)], intermediate (1,000–1,500 AUC), or low (400–1,000 AUC) satiety, respectively.

### Test Foods

The three types of plant-based foods involved were apple (Royal Gala variety), banana (Cavendish variety), and avocado (Hass variety). The nutrient composition for each of these foods has been reported (30). The food was cut into approximately 3 mm slices and served in 100 g portions in a covered plastic container. There was no limit to how many portions were available to allow participants to achieve comfortable fullness. The three plant-based foods were selected as examples of different sources of nutrient energy, such as soluble sugars for apple, starch for banana, and lipids for avocado.

### Satiety Measurement

Participants' fullness ratings were based on self-evaluation after the meal until 150 min through a 20 cm labeled magnitude scale (LMS) (33) due to the linguistically diverse population of participants. The LMS scale questionnaire was on printed paper. Participants were required to complete the questionnaire after leaving the lab for the last fullness measurement (at 150 min) and return the questionnaire at the next visit. Fullness ratings were quantified by measuring the distance (cm) between the greatest imaginable hunger marker and the marked point by participants on the scale. The distance is defined as perceived fullness at that time point. The total area under the curve (AUC) of the perceived fullness as a function of time up to 150 min was defined as satiety (3). The low, intermediate, and high satiety perceiving groups were defined based on the statistical spread of the data distribution. The low perceiving group corresponded with the lowest 25% of responders, the high perceiving group was the top 25% and the intermediate group represented the middle 50%. The statistical calculation used XLSTAT software.

### Body Composition Measurements

Body mass and volume, fat rate and fat mass, and fat free mass, were measured by air displacement plethysmography (BodPod, Life Measurement Inc. USA) following the manufacturer's instructions and using the Siri equation (34). Standing height (bare foot) was measured *via* a stadiometer (Leicester height measure, UK). Body density, body surface area (BSA) and total energy expenditure (TEE) were calculated and estimated using the BodPod. Body mass index (BMI) was calculated *via* the standard equation (body mass divided by the square of the height) and expressed in units of kg^*^m^−2.^ Body composition of participants was measured in a single session for each individual in the 2 weeks after the satiety sensory experiment.

### Human Metabolic Factors Measurements

Resting metabolic rate (RMR), respiratory quotient (RQ), and heart rate (HR) were measured by Indirect Calorimetry (TrueOne 2400 metabolic measurement system, Parvo Medics, US). The RMR and RQ were calculated using the device software according to the Weir equation (35). The energy (kilojoules) burning ratio from carbohydrate (K CHO) and fat (K FAT) during participants' resting status were estimated by the device software. Human metabolic factors for each of the participants were measured in the same session as body composition in the 2 weeks after the satiety sensory experiment.

### Oral Physiology

Participants' resting saliva flow rate, fungiform papillae density of the tongue and oral processing behavior (including oral processing time and number of chews) were measured as described in (31). Participants' maximum mouth water holding capacity volume was also measured. The mouth water holding capacity is a typical oral physiology measurement and associated with oral flavor perception (36, 37), which might be potential associated with satiety. So, this study considered it as a potential factor relating with satiety. A glass full of water, a plastic straw and a pre-weighed cup were provided for participants. Participants were instructed to suction as much water as they could hold in the mouth cavity through the straw. Then they expectorated all liquid into the empty pre-weighed cup and the weight of the cup measured. Mouth volume was reported in milliliters based on an assumed density of both water and saliva of 1.0 g/mL. Three replicates were collected, and the maximum volume measured was defined as the mouth volume.

### Psychology Factors

Participants' big five personality traits (openness, conscientiousness, extraversion, agreeableness, and neuroticism) (32) and food neophobic score (38) were measured by questionnaires. Eating behavior was measured using a questionnaire (Dutch Eating Behavior Questionnaire) that included 10 questions targeting restrained and external eating and 13 questions targeting emotional eating (39, 40).

### Data Analysis

Partial least squares (PLS) regression models were developed using variables described in Figure 2 [independent variables (X)] and satiety as dependent variable (Y). The Unscrambler software (The Unscrambler 11 software, Camo Analytics, Oslo, Norway) was used to develop the PLS regression models. Prior to modeling, independent variables (X) were standardized using 1/STD (STD: standard deviation). PLS regression models were developed using cross validation (leaving one out). The calibration models were evaluated using the coefficient of correlation (R), coefficient of determination in cross validation (R^2^) and the standard error in cross validation (SECV) (41–43). The number of samples were 63 (21 participants ^*^ 3 food types) for the PLS regression models.

The analysis of variance (ANOVA) and Pearson correlation statistical analysis was conducted in Microsoft Excel and XLSTAT software (Xlstat-Sensory 2021, Addinsoft, France). Satiety, psychological and oral physiological factors were based on 52 participants, whereas the body compositions and metabolic factors were based on 21 participants.

## Results

Table 1 shows the mean, range, SD and CV for satiety measured in the different groups used to develop the PLS models. The highest CV was observed for the male participants (31%) while the lowest was for the old participants (19%). The variance for both starting point fullness and the satiety AUC between food types were analyzed, however no significant differences were observed. This was a reason for analyzing the data together not separately for each food type. Based on their responses and grouping patterns across food types, individuals were classified as low, intermediate, or high satiety perceiving groups, defined as those individuals that report high (1,500–2,500 AUC), intermediate (1,000–1,500 AUC), or low (400–1,000 AUC) satiety, respectively. The PLS calibration statistics for the prediction of satiety using all variables (foods + psychology + physiology), psychology + physiology, and only physiology factors are reported in Table 2.

**Table 1:** Overall satiety statistics for different participant groups.

	**Number of observations**	**Min**	**Max**	**Mean**	**SD**	**CV (%)**
All	63	423.0	2,037.0	1,318.9	354.7	27
Old	17	971.3	2,008.3	1,532.9	293.2	19
Young	44	525.8	2,037.0	1,247.5	322.1	26
Male	36	423.0	2,037.0	1,263.8	392.0	31
Female	27	682.5	2,008.3	1,392.3	288.8	21
Intermediate satiety	36	1,031.5	1,510.8	1,308.8	139.7	11
Intermediate + high satiety	52	1,031.5	2,037.0	1,440.2	248.3	17
Low + intermediate satiety	47	423.0	1,510.8	1,176.9	281.0	24

*Min, minimum; Max, maximum; SD, standard deviation; CV, coefficient of variation (SD/mean X 100). Low, intermediate, and high satiety categories were defined as those individuals that perceive high (1,500–2,500 AUC), intermediate (1,000 − 1,500 AUC), and low (400–1,000 AUC) satiety, respectively*.

**Table 2:** Partial least squares regression statistics for the prediction of satiety using different combinations of variables.

	**N**	**LV**	**SECV**	**R**	**R^2^**	**Bias**	**Slope**
**Comparisons of variables**							
Food + Physiology + psychology	63	6	270.6	0.66	0.44	−0.40	0.59
Physiology + psychology	63	6	266.6	0.68	0.46	−0.47	0.57
Physiology	63	6	301.6	0.55	0.30	−3.00	0.40
**Comparisons of age groups**							
Old (age > 47 years)	17	2	247.4	0.61	0.37	1.93	0.45
Young (age from 18 to 46 years)	44	2	242.2	0.68	0.46	2.20	0.50
**Comparisons of satiety perception phenotype groups**							
Intermediate satiety	36	7	107.1	0.69	0.47	−4.03	0.64
Intermediate + high satiety	52	6	213.3	0.58	0.34	−3.02	0.49
Low + intermediate satiety	47	5	235.9	0.59	0.35	−3.07	0.48
**Comparisons based on gender**							
Male	36	3	300.5	0.66	0.44	1.34	0.57
Female	27	3	230.9	0.64	0.41	−4.56	0.49

*N, the number of samples; R, the correlation coefficient for validation; R^2^, the coefficient of determination for validation; SECV, the standard error in cross validation; LV, latent variable*.

The PLS model developed using psychology and physiology as input variables explained 68% (R = 0.68) of satiety, while the combination of all variables (food + physiology + psychology) explained 66%, i.e., inclusion of food variables did not improve the PLS calibration statistics for the prediction of satiety. Although this may suggest that food related variables (e.g., nutrient composition, food type, and energy density) do not contribute to explaining satiety, it is more likely that this reflects the fact that there were only three food types studied in this experiment. It is also possible that this is associated with the satiety experiment starting point (the same fullness level rather than the same food quantity or energy preload as in most satiety studies) reducing the effect of different food types. The PLS models developed using only the physiology variables explained 55% of the variance. This study has therefore shown partial interpretation of the relations between factors (involving food, human physiology, and psychology) and satiety. However, the interpretation of satiety was around half for each type of variable, suggesting that multiple variables (potentially including others not studied here) are needed to explain the observed variation in satiety. These may include food features (e.g., texture, flavor, structure) (36, 44) and human factors (e.g., digestive features, gastric emptying, nutrients absorption, and other psychological features) (45, 46) in addition to those measured in the current experiment.

**Figure 3:**
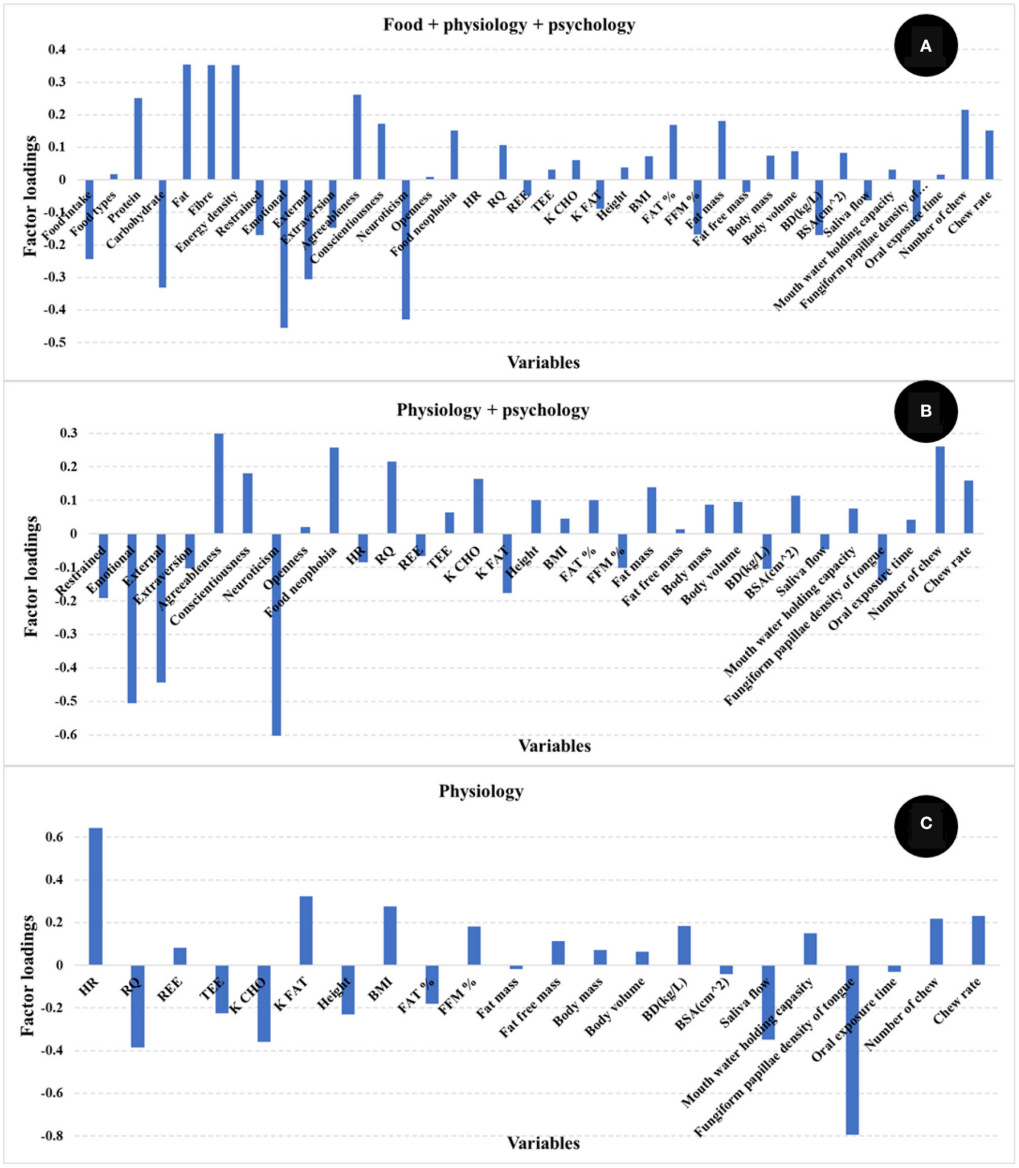
Loadings for PLS regression models comparing the different independent variables [**(A)** food + physiology +psychology, **(B)** physiology + psychology, **(C)** only physiology]. The factor loadings indicate the variables' contribution to satiety.

The number of PLS factors is derived from the cross-validation method where the optimum number of latent variables (LV) is determined by the lowest number of factors giving the minimum value of the standard error in cross validation. The addition of more PLS factors beyond this sweet spot, would not improve the amount of variance explained by the model. In this study, the most important loadings used by the PLS calibration models are shown in Figure 3. Examination of the loadings allows identification of variables or combinations of variables that contribute to explaining satiety. Based on the loadings reported in Figure 3, variables identified as most influencing satiety were metabolic factors [including RQ, and nutrient type used for energy (K CHO and K FAT)] and oral physiology variables (number of chews, chew rate, fungiform papillae density of tongue). Variables associated with personality or psychology traits such as agreeableness and conscientiousness contribute positively while neuroticism contributed negatively to explain satiety. In addition, variables such as emotional, external, and restrained eating (from Dutch eating behavior questionnaire) contributed negatively while food neophobia contributed positively to explain satiety. It has been reported that an increase in oral processing (e.g., bite size, chewing) resulted in increases in both satiety sensation and the timespan for satiety related hormones (e.g., CCK, GLP-1) (12, 14, 15). Moreover, Lasschuijt and others (12) reported that relations between oral processing and satiety were associated with food oral sensory exposure and perception. This is consistent with our result that oral processing and perception (fungiform papillae density of tongue) were key factors contributing to satiety (Figure 3) (12, 47). Other studies (19–21) have reported that personality traits and eating behavior (Dutch eating behavior questionnaire) were associated with food choice, food intake, and body weight management. High conscientiousness and agreeableness, and low neuroticism were mainly associated with self-discipline and suggested the inhibition of eating and more consumption of healthy food (e.g., fruits, vegetables) (20, 21). Additionally, emotional and external eating behavior were positively associated with neuroticism, lower conscientiousness, and lower extraversion, while restrained eating was related to high self-control (e.g., higher conscientiousness and lower neuroticism) (21). Our results (Figure 3) indicate that high self-discipline related to psychological factors including conscientiousness, agreeableness, and food neophobia positively contributed to satiety, while low self-discipline related factors like neuroticism, emotional, and external eating contributed negatively to satiety.

**Figure 4:**
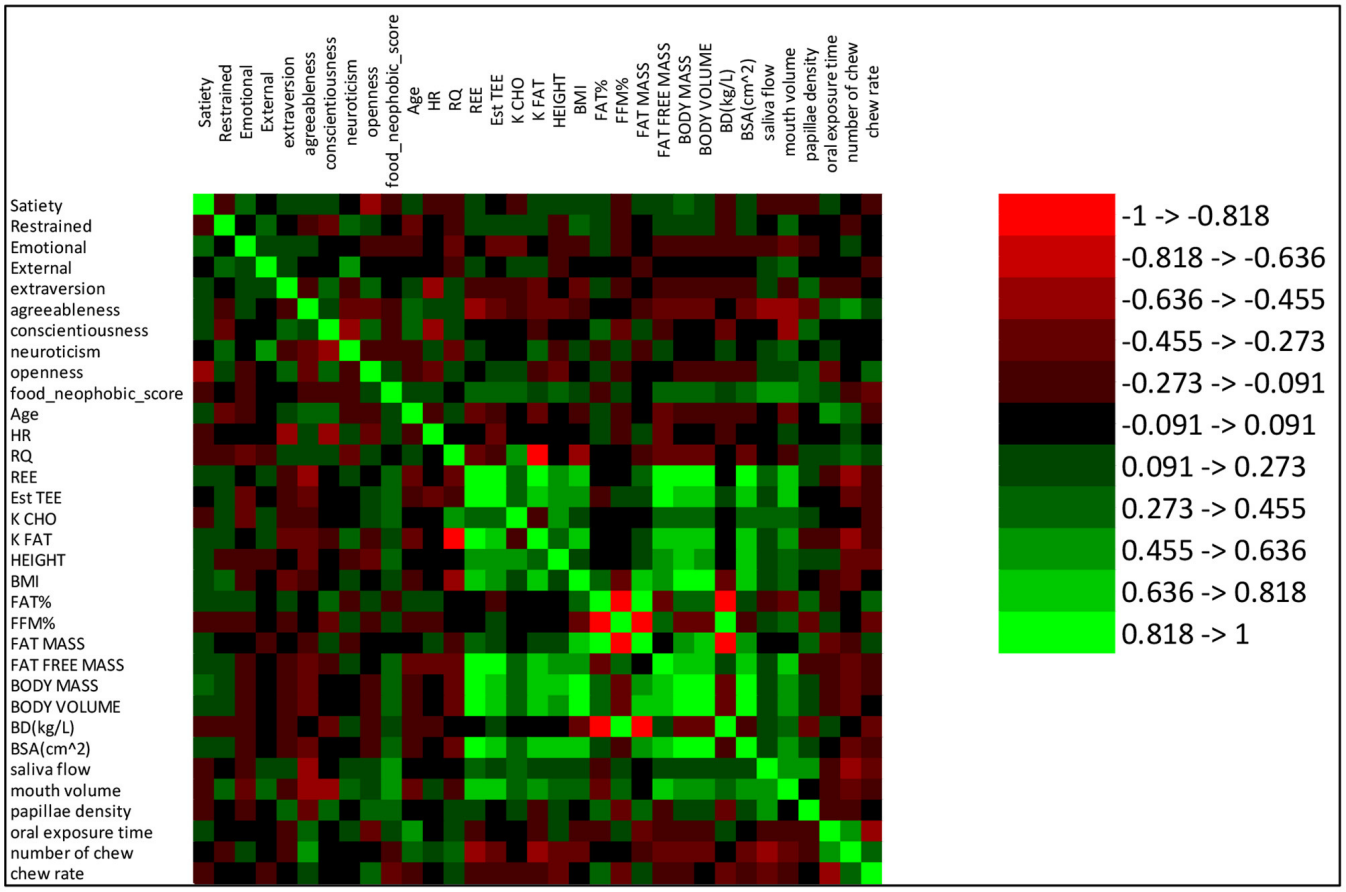
Correlations between measured variables. The color scheme number reference is on the right side of the figure. Red and green indicate negative and positive correlations, respectively.

This analysis of PLS model loadings shows that several human factors functioned in combination to explain nearly half of the satiety response. The identified human factors (including metabolic factors, oral physiology, personality and eating behavior pattern) were low in correlations (lower than 0.50) between each other (Figure 4). In addition, the correlations between each human factor and satiety were low (almost all around 0.25, Figure 4). The results of correlation analysis showed each single factor had only low correlation with satiety, and therefore a rationale for the further PLS regression modeling, which showed how the factors including human physiology and psychology combined in contributing to satiety. This demonstrates the importance of interactions between physiological and psychological factors in explaining individual variation in satiety responses.

### Gender Effects on Satiety

**Figure 5:**
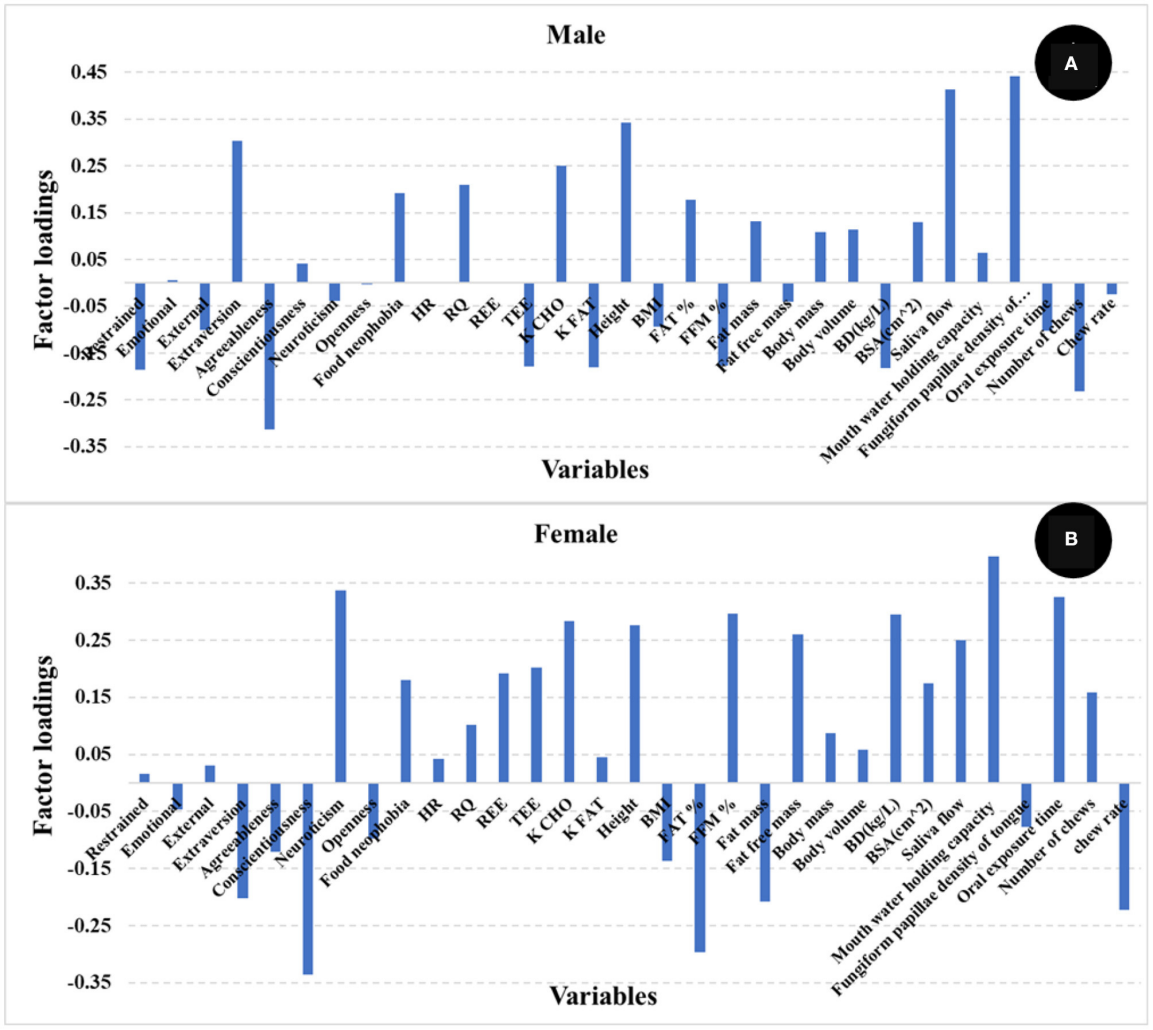
Loadings for PLS regression models comparing male **(A)** and female **(B)** subjects.

It has been reported by other authors that gender might contribute to explaining satiety (22). To evaluate the effect of gender on satiety in this study, PLS models were developed for male and female participants separately. Table 2 shows the R and SECV for the prediction of satiety using male (R 0.66, and SECV 300.5 AUC) and female (R 0.64, and SECV 230.9 AUC) participants, respectively. The loadings used by the PLS model are reported in Figure 5. It was observed that the PLS loadings used by each model were different particularly in psychology, oral sensitivity & processing, and body composition. This may indicate that different variables or the combination of them contribute differently depending on the gender group used to develop the PLS models. These results agree with those studies that reported that gender influences satiety, although no significant differences on subjective satiety were reported (22, 48, 49). No statistically significant differences in satiety were found with gender in this study either. However, both the pattern and contribution of human factors to satiety were different between male and female participants.

### Age Effects on Satiety

**Figure 6:**
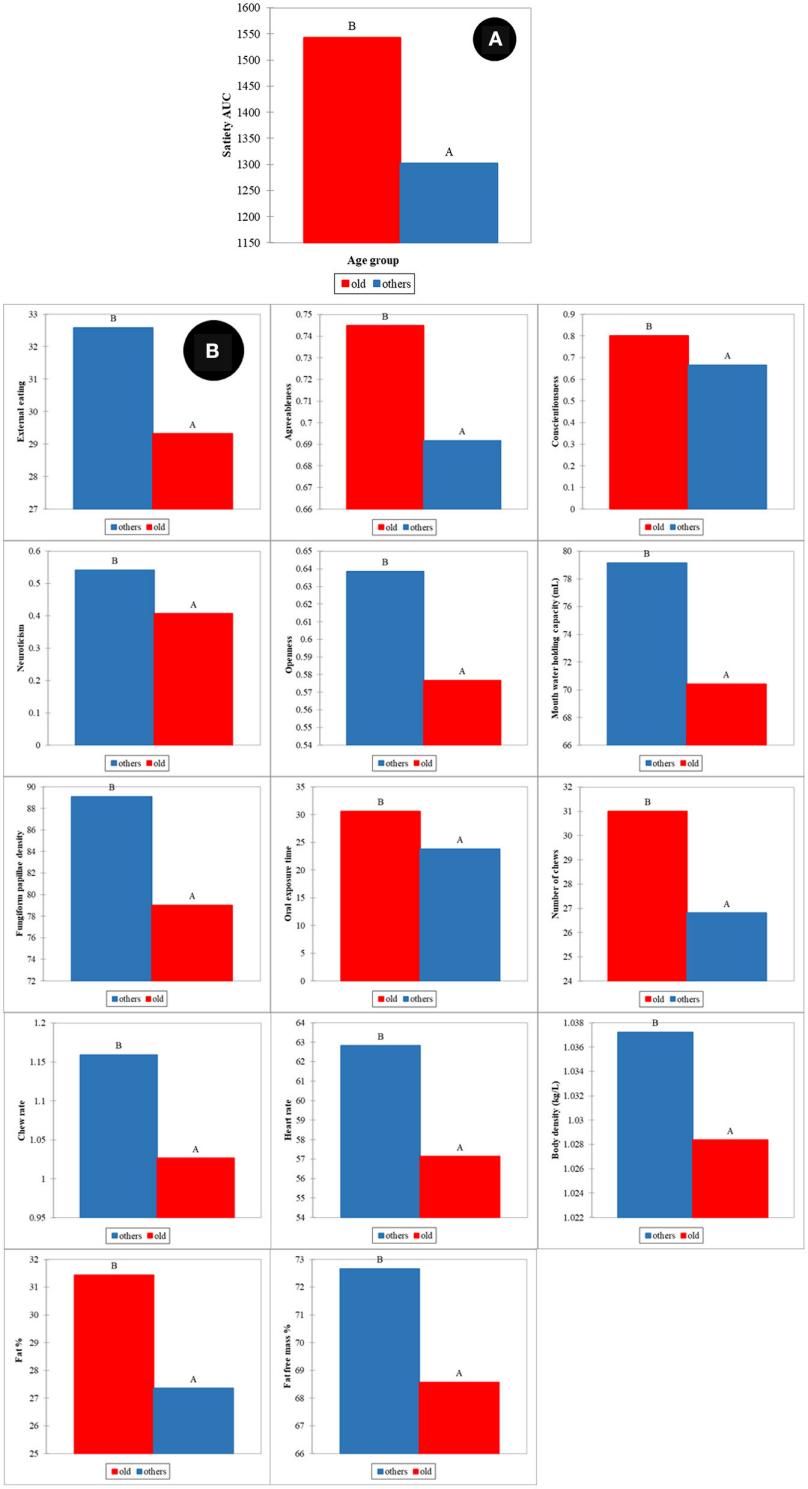
Age effects on satiety **(A)** and human factors **(B)**. All mean differences between age groups are significant at the 0.05 level and labeled with different letters.

Similar to gender, the effect of age on satiety has also been reported by different authors (22, 24, 26). In our study, two groups were defined as old (participants age over 46 years) and young (participants age in the range 18–46 years). The ANOVA showed that statistically significant differences between old and young participants were found for satiety (*p* < 0.001) (Figure 6A). Variables associated with human factors had statistically significant differences between old and young participants (Figure 6B). External eating behavior (*p* < 0.001), agreeableness (*p* < 0.05), conscientiousness (*p* < 0.001), neuroticism (*p* < 0.05) and openness (*p* < 0.001) were significant differences in psychology between young and old participants. Old participants had statistically significant lower mouth water holding capacity (*p* < 0.001), fungiform papillae density of tongue (*p* < 0.05), and chew rates (*p* < 0.05), together with higher oral processing time (*p* < 0.001) and number of chews (*p* < 0.05). Old participants were higher in body fat % (*p* < 0.05) and lower in fat free mass (FFM) %, body density, and heart rate (HR) (*p* < 0.05).

**Figure 7:**
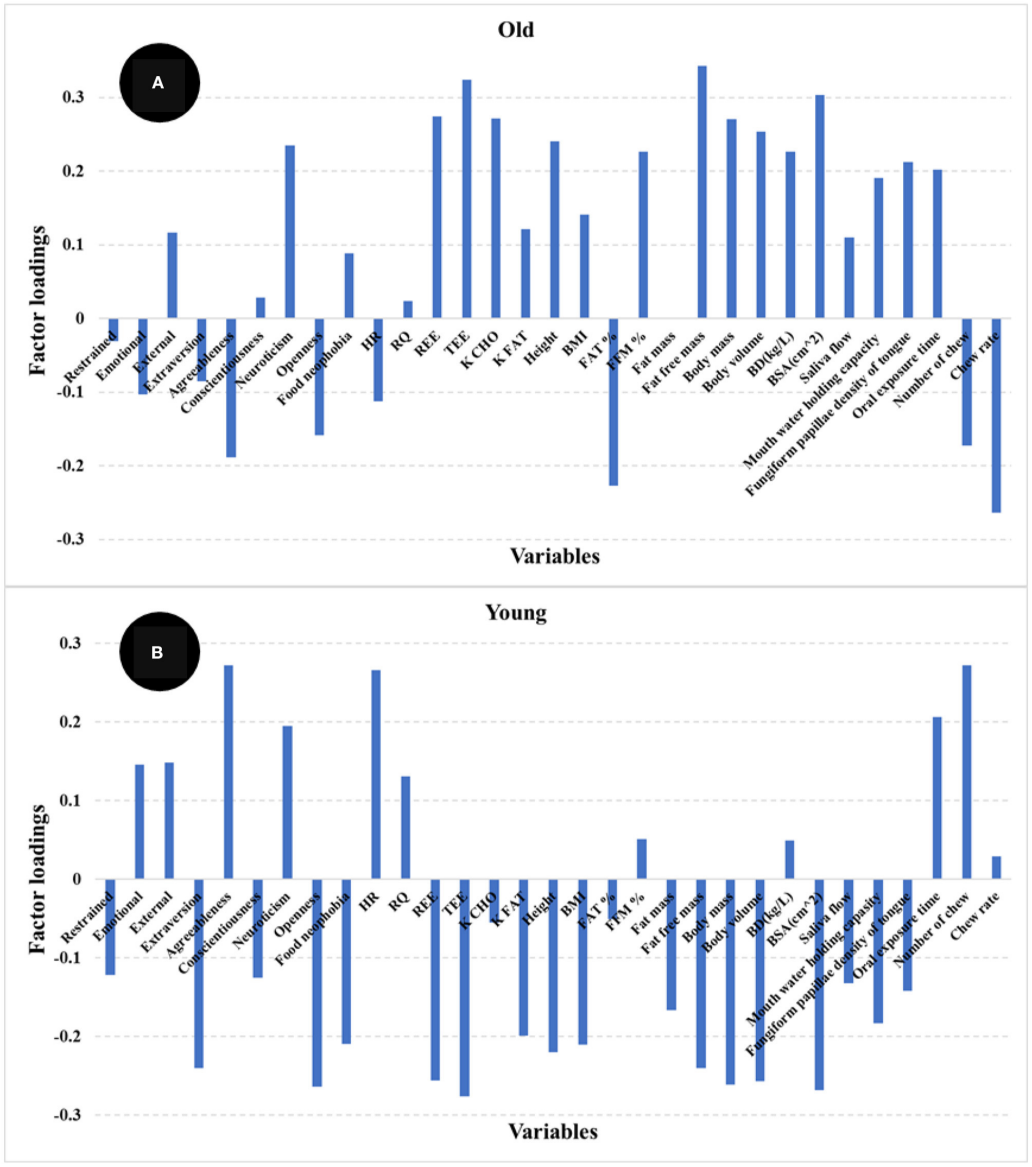
Loadings for PLS regression models comparing older (>46 years) **(A)** and younger (<46 years) **(B)** participants.

The PLS regression statistics for the prediction of satiety using different age groups are reported in Table 2. The R and SECV for the prediction of satiety were 0.61 and 247.4 AUC and 0.67 and 242.2 AUC for the old and young participants, respectively. The highest positive loadings for the old group (Figure 7A) were neuroticism, metabolic factors (REE, TEE, and K CHO), body composition (fat free mass, FFM%, body mass, body volume, and BSA) and oral physiology (fungiform papillae density, and oral processing time). The greatest negative loadings were agreeableness, fat%, number of chews, and chew rate. In contrast, the highest positive loadings for the young group (Figure 7B) were agreeableness, neuroticism, HR, oral processing time, and number of chews, while highest negative loadings were extraversion, openness, food neophobia, metabolic factors (REE, and TEE), body composition (fat free mass, body mass, body volume and BSA), and fungiform papillae density of tongue. Overall, three psychological (agreeableness, conscientiousness, food neophobia) and almost all physiological factors (except RQ, Fat %, FFM %, and oral processing time) were the main variables contributing to explain satiety in both young and old participants but in different ways. These results indicate that differences between groups may be associated with age-related changes in body metabolism and composition, oral physiology, and psychology.

These results agreed in general with reports by other authors (22, 24–26). These authors highlighted that decrease in pleasantness (e.g., food hedonic and sensory perception), changes in metabolic factors, weakening of appetite-related hormone responses (e.g., CCK and ghrelin), as well as changes in psychological and social factors were potential explanations for the differences in appetite for elderly participants (24–26, 50, 51). Our results indicate that oral perception and processing capability decrease with age, while an increase in fat % and decreased fat free mass % body composition was observed for old participants. More importantly, these factors showed the highest loadings in the PLS models used to predict satiety. Statistically significant differences in personality and eating behavior pattern for the older participants were also observed and these factors showed high loadings in the PLS models for satiety prediction.

### Individual Characteristics Associated With Satiety Perception Phenotype Groups

**Figure 8:**
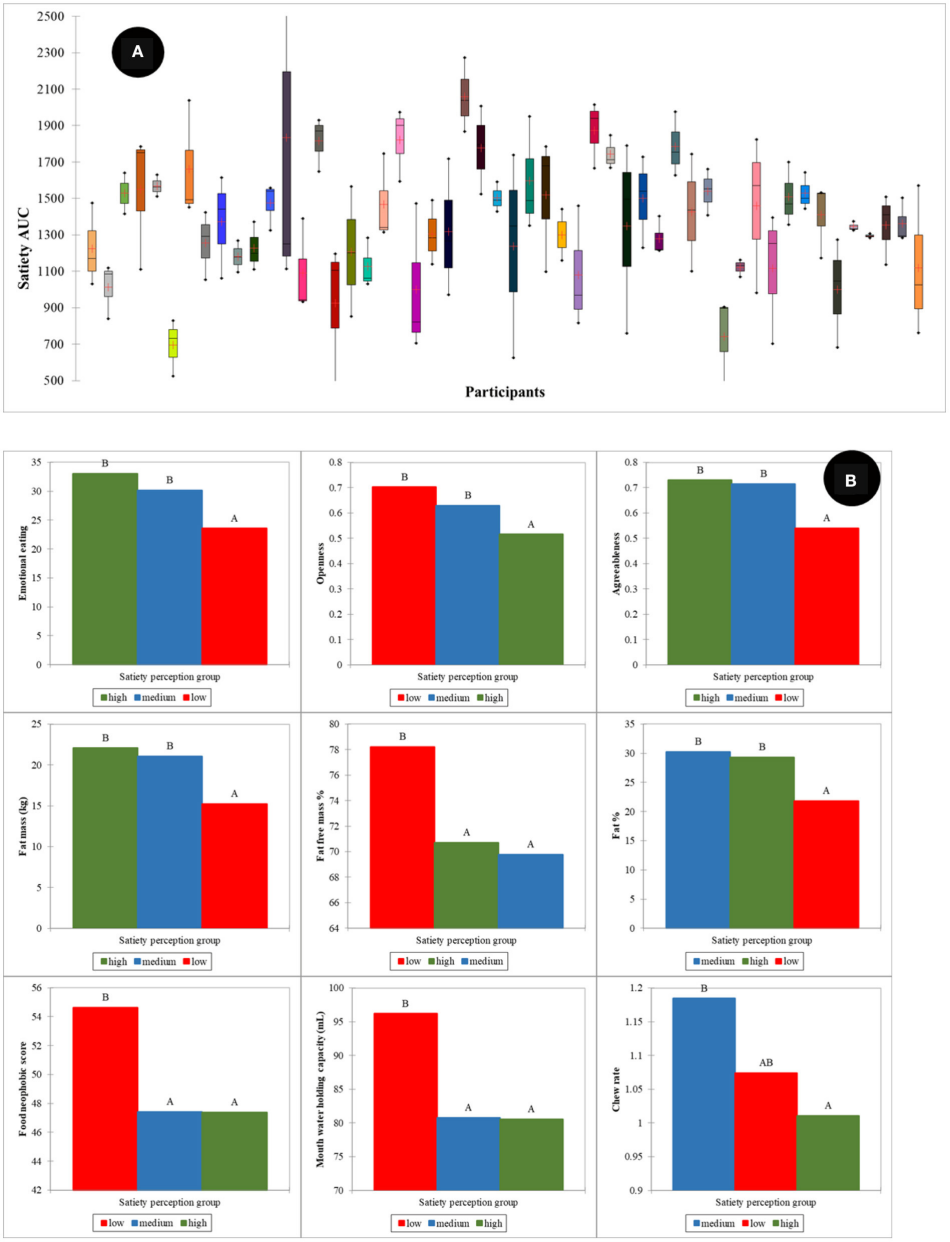
Individual variation in satiety perception **(A)**, human factors differences between satiety perception phenotype groups **(B)**. Low, intermediate, and high satiety categories were defined as those individuals that perceive high (1,500–2,500 AUC), intermediate (1,000–1,500 AUC), and low (400–1,000 AUC) satiety, respectively. Where the mean difference between groups is significant at the 0.05 level they are labeled with different letters. The results are summarized from all food types.

Large individual differences in reported satiety perception were observed as depicted in Figure 8A. To investigate the effect of these individual differences in calibration of reported satiety perception, three groups were defined. These groups were associated with those individuals that perceive high (1,500–2,500 AUC), intermediate (1,000 −1,500 AUC), and low (400–1,000 AUC) satiety, respectively. Most of the high satiety perception phenotype group were old participants. PLS models for the prediction of satiety were developed using these three groups. Due to the limited number of samples in both high and low satiety groups, individual PLS models for these groups were not possible. Therefore, PLS regression models were developed as follows for all participants: intermediate, combining low and intermediate, and combining high and intermediate satiety groups. The R and SECV obtained for the prediction of satiety were 0.69 (107.1 AUC), 0.58 (265.2 AUC), and 0.59 (213.3 AUC) for the intermediate, combination of high and intermediate, and low and intermediate groups, respectively (Table 2).

**Figure 9:**
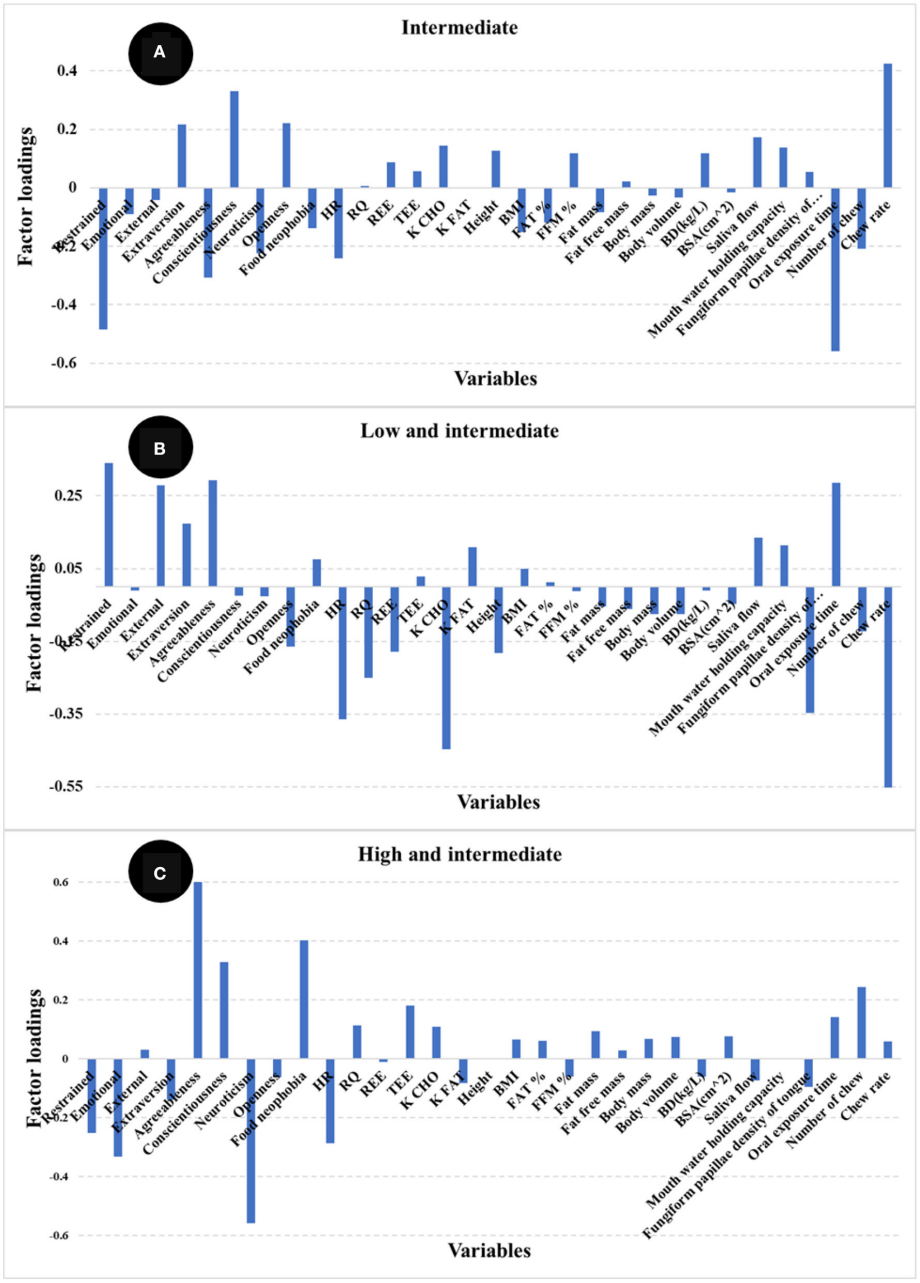
Loadings for PLS regression models comparing satiety phenotypes [**(A)** intermediate satiety phenotype, **(B)**. low and intermediate satiety phenotype, and **(C)** high and intermediate satiety phenotype].

The highest positive loadings for the intermediate satiety group (Figure 9A) were chew rate, conscientiousness, extraversion, and openness, whereas the negative loadings were oral processing time, restrained eating, agreeableness, neuroticism, and HR. In contrast, the highest loadings for the low and intermediate satiety group (Figure 9B) were restrained and external eating, agreeableness, and oral processing time (positive), together with chew rate, fungiform papillae density of tongue, HR, RQ, and K CHO (negative). The high and positive loadings for the high and intermediate satiety group (Figure 9C) were agreeableness, conscientiousness, food neophobia, and number of chews, whereas restrained and emotional eating, neuroticism, and HR (negative). When comparing loadings between the models, large differences in the main variables contributing to the different satiety phenotypes were observed.

The ANOVA showed statistically significant differences in physiological and psychological factors between the different satiety phenotypes (Figure 8B). The high satiety group had significantly lower openness (*p* < 0.001) and chew rate (*p* < 0.05) than the other two groups. For the low satiety group, emotional eating (*p* < 0.01) and agreeableness (*p* < 0.001) were significantly lower while food neophobia significantly higher than the others. In physiology, the low satiety group had significantly lower fat% & fat mass (*p* < 0.01) and higher mouth water holding capacity (*p* < 0.05) than other groups.

The prevalence of low satiety phenotype groups was also reported by Drapeau et al. (27). These authors reported that low satiety phenotypes might be associated with stress and anxiety. In our results, significant differences in physiological and psychological factors were also found between different satiety phenotype groups. For example, the low satiety phenotype group had significantly lower fat % and fat mass in body composition. This may be associated with lower blood cortisol response to the meal in the low satiety phenotype group (27, 52). Moreover, other studies report that low satiety phenotype is associated with greater preference for high energy foods, desire to eat, and lower craving control (53). In our findings, the low satiety phenotype had significantly higher food neophobia and higher mouth water holding capacity, which might be associated with high food preference and desire to eat (37, 54). The lower craving control might be associated with the lower agreeableness score.

## Discussion

Using an experimental design that instructed participants to eat until comfortably full, the subsequent reported satiety feelings could be partially (46%) explained by a combination of measured physiological and psychological variables. Food intake, food type, and nutrient composition contributed limited information to explaining satiety in our study, but this may simply be a reflection of the limited number of food types (three) studied. Another possibility that is worthy of future study is that a satiety experiment starting at the same fullness level rather than the same food quantity or energy preload may reduce food effects on satiety differences. From a PLS model, the most important variables included metabolic factors [RQ and body energy usage type (e.g., carbohydrate or fat)], oral physiology (fungiform papillae density of tongue, number of chews, and chew rate), personality traits (agreeableness, conscientiousness, and neuroticism), and eating behavior pattern (e.g., emotional, external, and restrained eating). This suggests that interventions targeting these identified top attributes (except for the unmanageable attributes), or a combination of attributes could be used to modulate satiety feelings.

Influences on reported satiety related to individual variance (in terms of age, gender, and satiety phenotypes) were also investigated. Gender had only a slight overall effect on satiety, but the human factors contributing to satiety the most were different between males and females, particularly in psychology, oral sensitivity & processing, and body composition. Older people had significantly higher satiety feelings than young, related with significant differences in (faded) oral physiology, (increased) fat in body composition, and mature psychological characteristics (e.g., lower external eating, lower neuroticism, higher agreeableness, and higher conscientiousness). Different satiety phenotypes were not only based on individual variation in subjective feelings, but had significant correlations with body fat, oral physiology, personality type, food neophobia and eating behavior patterns. It is therefore important to consider individual variations in relation to age and satiety phenotype in research and food product design to control these otherwise confounding effects.

This study has shown that in order to understand the factors that control perceived satiety, multiple domains (physiology, psychology, food) need to be considered, and that there is marked individual variations partly associated with age and gender. Future studies aimed at modulating satiety need to take account of these diverse factors in proposing approaches to food intake management.

Further, for holistic studies focusing on the study of satiety responses of human to foods, it is important to consider the effects of both individual and food variance. An adequate number of participants based on balancing the individual variations in terms of age, gender, and satiety perception phenotype need to be determined. Unfortunately, the number of samples in this study were influenced by the COVID-19 pandemic. Moreover, this study showed that multivariate analysis (e.g., PLS regression) is suitable to interpret the relationships between multiple factors and human satiety. In the future, it is recommended that studies with more variables and food types or diets be conducted to provide a more holistic view of human-food interaction in terms of satiety response.

## Data Availability Statement

The original contributions presented in the study are included in the article/supplementary materials, further inquiries can be directed to the corresponding author.

## Ethics Statement

The studies involving human participants were reviewed and approved by the Sub-Committee for Human Research Ethics of the University of Queensland Science (approval number: 2019002688). The patients/participants provided their written informed consent to participate in this study.

## Author Contributions

HS, MG, DC, and DN: conceptualization. HS and DN: methodology. DC and DN: formal analysis. DN: investigation and writing-original draft preparation. MG, DC, and HS: writing-review and editing. All authors have read and agreed to the published version of the manuscript.

## Funding

The authors acknowledge funding from Hort Innovation (Australia).

## Conflict of Interest

The authors declare that the research was conducted in the absence of any commercial or financial relationships that could be construed as a potential conflict of interest.

## Publisher's Note

All claims expressed in this article are solely those of the authors and do not necessarily represent those of their affiliated organizations, or those of the publisher, the editors and the reviewers. Any product that may be evaluated in this article, or claim that may be made by its manufacturer, is not guaranteed or endorsed by the publisher.
